# Practical Suggestions to Young Physicians

**Published:** 1854-05

**Authors:** T. P. Atkinson

**Affiliations:** Danville, Va.


### *Practical Suggestions to Young Physicians.*

BY T. P. ATKINSON, M. D., DANVILLE, VA.

We know not that we can, in any way, render a greater service to the young and inexperienced members of our profession, than by offering the following suggestions as to the proper rules to be observed in adapting and proportioning the medicines which they use in the treatment of disease. We well remember the embarrassment which we sometimes felt in the early days of our professional career, in consequence of the failure of the remedies administered to meet what we regarded the plain indications of the case. Subsequent experience has taught us not only that the error was in us, but how, to our own comfort as well as to the relief of our patients, we might have avoided that embarrassment. We shall be compensated, in some measure, for the pain to which we were subjected, if our experience shall be the means of saving others from like error and like suffering.

The common fault of all novitiates in the practice is, that they are what is familiarly known as *routine practitioners:* that is, they *follow*

*too closely the directions of books,* without changing the medicines and regulating their doses *according* to the *varying symptoms and exegencies of each particular case.* When called to the bedside of a patient, after having made himself acquainted with his feelings, together with all the circumstances preceding and attending the attack, they examine his skin, pulse, tongue, secretions, &c. Possessed of the important information thus derived, they too frequently set about thinking what *the disease* is *t rmed,* and what the *treatment* recommended in that affection, by their *text book on the practice,* or by some other work *specially brought to their notice by their instructor.* Having satisfied themselves on these points, they are at no loss for the remedy. It may be that an emetic or cathartic is promptly administered ; but instead of operating as was expected, it makes no sensible impression whatever, or it produces a result the opposite of what was desired.

Disappointed and discomfited, the young and timid practitioner consults another favorite author, whose prescription proves as inopetive or as injurious as those already administered. In this dilemma he either gives up in despair, and abandons his patient to his fate, or he calls in a consulting physician whose experience enables him to detect the error, and who, by continuing the remedy first used, in increased or diminished doses or in new combinations, or by substituting some other of directly opposite qualities, relieves both the doctor and his patient.

We will suppose that the case under treatment is one of bilious remittent fever, accompanied with torpor or with congestion of the liver. Calomel has been administered in the usual doses ; but instead of unlocking and relieving the suffering organ, the bowels are stimulated to an inordinate degree, and copious watery evacuations threaten to prostrate the patient. The experienced and sagacious practitioner who has been called in consultation, instead of throwing aside that great regulator of the secretions, doubles or quadruples the dose, combining it at the same time with opium in some form, and thus not only promptly arrests the serous discharges from the bowels, but causes the liver to pour out healthy bile in proper quantity, which prepares the patient for the quinine, the timely administration of which saves a valuable life, and most forcibly demonstrates the importance of adapting the remedy, both in character and quantity to the circumstances of the case.

If the immortal Rush (whom I have been sometimes disgusted to hear reviled by the unfledged disciples of Broussais) had rendered no other service to mankind than by the simple inculcation of the important principle that the only philosophical and safe rule in prescribing medicines is to disregard the name of the disease and be guided by the symptoms as they present themselves. he would have deserved to be regarded as one of the greatest benefactors of his race as well as one of the greatest reformers in medicine who ever moved in the tide of time. I am satisfied that a large portion of all the deaths which have ever occurred from disease of any character, has resulted from a neglect of this all important principle. The fact is that no individual, whatever his talents or his learning, can be a successful practitioner if he overlook or disregard this fundamental truth in medicine.

The first object to be accomplished on visiting a patient should be to form a correct diagnosis of the case. To do this the physician should notice not only the symptoms as they appear on his inspection, but he should enquire minutely into the antecedents of the case, as well as into the habits, predispositions, idiosyncracies, &c. of the patient. Having satisfied himself thus of the true condition of the various organs implicated, and of the indications to be effected, he is prepared to select the remedy most proper to be administered. With his opinion formed after this careful consideration of all the circumstances of the case, and with his course thus marked out, he should not be turned from his purpose by "light and transient causes."

There should be no vacillating, no "shifting and dodging" from one medicine to another, unless a change shall become necessary by the occurrence of some new symptom, or by the discovery of one which had been previously overlooked. It often happens that the physician has taken a proper view of the case under treatment, and has been for hours or days administering the proper medicine; and yet he may have seen no improvement in the condition of his patient, but rather an aggravation of the disease. Now we would warn the inexperienced and timid practitioner against hastily determining that, because of this discouraging state of things, he has been giving the wrong remedy. Let him rather carefully re-examine the condition of the sufferer, and review the course of reasoning by which he was led to form his diagnosis. Above all, let him consider the circumstances which may cause the case under treatment to differ from ordinary cases of the same disease, either in character or intensity; and he will often find that by diminishing or increasing the quantity of his remedies, or by giving them in new combinations, or, as sometimes happens, by administering them in another vehicle from that first used, the disease will be arrested and his patient restored to health.

In many cases much, we will go farther and say all, depends on the quantity and combination of the medicines administered. In croup, for example, it often requires as much tartar emetic to excite vomiting as would be hazardous to give to half a dozen children in health. So in bilious colic and its appropriate remedies, not to mention other diseases and other medicines, (for the principle holds good in all,) we are forced to give opium in combination with calomel, in five or ten times the usual quantity. This article, it is well known, is commonly given in doses of a grain or two, to relieve pain or to arrest purging; and yet given in that quantity in violent bilious colic, attended with severe spasm, it would produce no good effect; while, if it be administered in greatly increased doses, it will not only relieve the pain, but by removing the spasm on which the pain mainly depends, it will greatly favor the purgative effect of the calomel. These cases (and such might be multiplied almost indefinitely) are sufficient to show the importance of the principle which we would inculcate, namely, that no one can be a successful or a safe practitioner who does not adapt his remedies and graduate his doses to the varying circumstances of each case in which he administers them.

The truth is, that the word "dose" is altogether a relative term.

When, for instance, it is laid down in treatises on Materia Medica, that from one grain to three grains of tartar emetic is a dose, it is by no means intended to convey the idea that one grain is the minimum and three grains the maximum quantity in which that medicine should be administered. It means only, that in a majority of cases, vomiting will be excited by that quantity taken into a healthy stomach. The regulation of the dose in any given case, must, of necessity, be left to the judgment of the practitioner, who should be guided in all instances, by the circumstances under which it is used. And so of opium and indeed of all other medicines. We know that from one to two grains of this drug is said to be a dose; and yet it sometimes happens in mania and some other affections, that more than twenty times the larger quantity mentioned above is required to calm the nerves and control the inordinate mental excitement of the sufferer.

The same principle is applicable to blood letting. When the abstraction of from twelve to twenty-four ounces of blood is recommended in the treatment of inflammation of any vital organ, it is not meant that not more than the last mentioned quantity shall be drawn in any such case, for we frequently have to abstract more. We should always go for the effect and not for any given quantity. The exact guage must, as in the case of medicines, be left to the judgment of the practitioner, who should regulate it according to the symptoms before him. We shall not attempt an enumeration of all the various causes which create the necessity for this variation of the character and quantity of medicines, but will allude to only a few of them. The practitioner in the northern section of our Union stands aghast when he hears of the hundred grain doses of calomel and the thirty grain doses of sulphate of quinine which are sometimes given in the bilious remittent and the continued fevers of the South; and he is incredulous as to the propriety of the treatment. But if he could witness the cases and the success of these remedies thus administered, he might become convinced that climate may exert such a controlling influence as fully to vindicate the propriety of the practice.

We have elsewhere spoken of the habits of the patient as a matter which should be carefully enquired into. We not unfrequently find that from long indulgence in the use of ardent spirits by our patients, we are inhibited from abstracting as much blood as would be required under other circumstances; and in such cases when stimulants are indicated, they have to be administered in larger quantities than would ordinarily be admissible. And so in those cases where the patient has indulged in the habitual use of opium in any form, much larger doses are required, and we have to be governed, in determining the quantity of that article to be given in any supposed case, altogether by the effect, taking care to give enough.

Before dismissing this branch of our subject, we would remark, that the young physician is not unfrequently beset, by the importunities of the patient or his friends, to give medicine, not only when none is required, but when it would be highly improper and injurious to do so. It often happens that the remedy which had been previously administered is acting as was expected, and the physician finds it proper to leave his patent without ordering any other medicine. It may be that the latter is restless and impatient, and may imagine (as we have often known to be the case) that nothing but the gulping down a dose of medicine every hour or two can relieve his sufferings and restore him to health. If, under these circumstances, the physician leave him without prescribing something, the individual considers himself neglected, and in all probability concludes that the doctor has mistaken the nature of his case. The inquietude and apprehension occasioned by this conviction may of themselves aggravate the disease, and thus injure the patient; or if he be not so alarmed, he is rendered dissatified with his physician, and perhaps dismisses him, and much to his own injury, calls in another, who, willing to humor him in his notion, or it may be, satisfied that his predecessor has erred, pours down dose after dose, which so interferes with the curative operation of nature then going on to the satisfaction of the dismissed physician, as to induce a new and fatal action, and thus to destroy the patient. Are we asked what the practitioner is to do in such an emergency? We reply that, as in all other instances, the character and quantity of the remedy should be determined by the exigencies of the case.

Satisfied that any interference with the curative action of the vis medicatrix naturæ, at that particular stage of the disease, would be improper and injurious, he should, to preserve the confidence and calm the apprehensions of the patient and his friends, direct some simple thing, such as a few drops of sweetened cochineal water, or a drop or two of sweet spirits of nitre diffused in a teaspoonful of water, or some other equally harmless article, to be given every four or six hours, until such time as some other and more active remedy may become proper. Let no one say that this would be practicing an unjustifiable deception; that it would be in fact "doing evil that good might come of it." We are not the advocates of any such jesuitical action as would be implied by such a rule of conduct. And we deny that the course advised would be obnoxious to such a charge.

That profound thinker and sound moralist, the immortal Lord Bacon, was of opinion that there are exceptional cases, in which "morality submits to a suspension of her rules in favor of her principles." We by no means admit that we are driven to this exception in justification of the practice above recommended. We do not regard the course advised as a departure from the strict rules of morality. It is no deception, because the harmless prescription recommended is not only a remedy, but the best remedy that can be administered under the circumstances of the case.

Again, there are certain predispositions which should never be disregarded in the administration of medicines. We have known persons so predisposed to hemorrhage from the lungs or to apoplexy or hernia, as to render it hazardous to give an active emetic. The same may be said of other affections and other remedies.

Furthermore, there are idiosyncracies which strongly forbid the use of particular remedies, which otherwise would be best adapted to the cases under treatment. There are many individuals who cannot take calomel in any quantity, (even the smallest) without being salivated. There are others who are immediately subjected to very violent attacks of dyspnœa, by even the slightest smelling of ipecac. We have a friend who cannot enter a room in which there is a vial of Dover's powder unstopped, without having his breathing painfully affected in an instant. Now in all such cases it would be the height of folly for the attending physician to overlook or disregard these peculiar circumstances. They all should be carefully weighed in forming a judgment as to the proper remedy to be used.

While on the subject of ipecac, we will remark, that we always give it in larger doses than any of the books recommend, if we desire to obtain its emetic effect; for this reason, that while it is a mild and gentle emetic, it frequently proves to be a troublesome cathartic, producing frequent watery discharges and irritating the mucous membrane of the intestines in no ordinary degree. To prevent this disagreeable result and to insure the emetic effect, we have been in the habit of giving about double the quantity usually advised by the books. It is only when given in doses of from ten to twenty grains, or thereabouts, or when in larger doses it fails to excite free vomiting, that we have found it apt to produce the unpleasant result above stated. In very minute doses, even without opium, it is frequently very efficient in arresting dysenteric discharges, by determining to the skin.

There is yet another consideration which should have no inconsiderable weight in regulating the character and quantity of the remedies used in the treatment of disease. It is one too which we are persuaded is not always sufficiently regarded, even by the experienced practitioner. We allude to the constitution of the patient as affected by color. We had not been long engaged in the practice of medicine before we were fully convinced that what will cure a white person of fever or other disease, will kill a negro suffering under a like affection. They do not require nor will their systems bear depletion of any kind, either by blood letting or purging, to the extent which is demanded in the case of white persons. Emetics, on the contrary, seem to be better adapted to them than to whites, and stimulants are much sooner called for in the treatment of their diseases. As the necessary result of this peculiarity, they are far less able than white persons to withstand the inroads of any disease of an inflammatory character, as they are much more liable to sink under attacks of typhoid or typhus fever. In the one case, because of their inability to bear up under the necessary depletion; and in the other, because of the tendency of their system to sink under the depressing influences of that fearful malady. In making out our prognosis, therefore, as well as in determining on the remedies and their proper quantities, we should not disregard the color of our patients.

There is only one other consideration to which we will advert in this article, and that is the importance of paying attention to the proper combination of medicines. An acquaintance with all the properties of every article of the materia medica will not qualify any one for prescribing them, unless he possess a knowledge of their relations to each other. Without this knowledge, the practitioner must grope his way in the dark, liable, on the exhibition of every compound, to become the unconscious, though not the innocent murderer of his patient—for he can never know whether he is administering an inert, a remedial or a poisonous mixture.

It is of the first importance, therefore, for the physician to know, not only the properties of each separate article which he prescribes, but, that he shall be fully acquainted with their relative affinities.

We will not stop to mention any of the numerous incompatible articles used as remedial agents ; but we would impress on the minds of our young brethren the vital importance of making themselves fully acquainted with them all, and of never combining medicines without special regard to their affiinities.

The same injunction will apply to the vehicles in which the medicines are administered. A syrup containing an acid of any kind, for example, is a very improper menstruum in which to mix certain powders which are in every day use, not only by the profession, but by mothers and nurses. How many "little innocents," who were the "darling of their parents," have fallen victims to ignorance on this subject, on the part of their doting mothers or fond nurses, we care not to know. We are satisfied, however, that their name is legion.

By way of impressing on the mind of the reader what we have written, we will sum it all up in the following brief expression : Never combine medicines without knowing and regarding their chemical affinities ; and in apportioning the doses of all remedies, give just as little as will do and just as much as is required.

